# Beech Leaf Disease Associated With Changes in Litter Decomposition and Fungal Communities

**DOI:** 10.1002/ece3.72563

**Published:** 2025-11-29

**Authors:** Brianna L. Shepherd, Sarah R. Carrino‐Kyker, David M. Jenkins, David J. Burke, Katharine L. Stuble

**Affiliations:** ^1^ The Holden Arboretum Kirtland Ohio USA

**Keywords:** American beech, carbon storage, decomposition, ecosystem function, forest health, fungal communities, leaf litter

## Abstract

Forest pests and diseases may indirectly drive critical shifts in rates of litter decomposition, ultimately impacting carbon and nutrient cycling. Beech leaf disease (BLD) is a rapidly emerging forest disease affecting American beech and driving significant changes in leaf traits and associated microbial communities—both critical drivers of decomposition. However, the extent to which these BLD‐driven changes may alter rates of litter decomposition remains unknown. Here, we explore rates of decomposition in BLD symptomatic and asymptomatic leaf litter using a field‐based litterbag experiment. In addition to monitoring rates of decomposition, we also explored differences in the associated fungal communities. We found that BLD‐symptomatic litter decomposed more quickly than asymptomatic litter. Furthermore, fungal communities differed in composition across symptomatic and asymptomatic leaf litter, with higher abundances of saprotrophic fungi found on symptomatic litter throughout the decomposition process. Given the dominance of American beech in many forested systems in northeastern North America, these shifts in litter decomposition rates driven by BLD may have important consequences for nutrient and carbon cycling in many deciduous forests.

## Graphical abstract

Less than 15 years ago beech leaf disease was identified as a newly emerging forest pest. There is still little known about the larger ecosystem impact. Our study is at the forefront of understanding how this newly emerging disease is impacting leaf litter decomposition and fungal communities in highly infested areas.

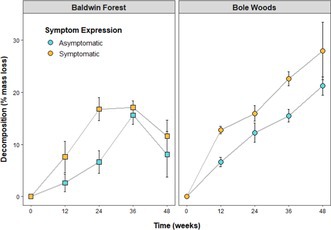

## 1 Introduction

Pests and pathogens threaten forest ecosystems worldwide, driving changes in species composition and altering ecological processes (Boyd et al. 2013; Dietze and Matthes 2014). Beech leaf disease (BLD) is an emerging forest disease impacting *Fagus grandifolia* (American beech) and other beech species (Colbert‐Pitts et al. 2025; Ewing et al. 2019). BLD was first discovered in northeastern Ohio, USA in 2012 and has since spread across 15 states in the northeastern United States and into Ontario, Canada (Cleveland Metroparks 2023). Within the disease's epicenter, mortality rates have been as high as 30% within the first decade of infestation (Shepherd et al. 2025). Given the status of American beech as a dominant tree species in deciduous forests throughout the region, BLD has the potential to dramatically reshape the composition and function of many forests.

BLD is caused by the foliar nematode *Litylenchus crenatae* spp. *Mccannii* (Carta et al. 2020). Leaves of infested trees are visually distinct, characterized by dark interveinal banding and crinkling of the leaf (Martin and Volk 2022; Ewing et al. 2019). Leaf buds may also be aborted before fully expanding into leaves (Ewing et al. 2019; Fearer et al. 2022). Infested leaves appear thicker, with increased spongy mesophyll, palisade layers, and abaxial epidermis (Fletcher et al. 2024; Vieira et al. 2023). As a result, the specific leaf area (SLA) decreases by upwards of 60% in crinkled symptomatic leaves relative to asymptomatic leaves. Total leaf nitrogen (N) increases with disease severity, with the highest leaf nitrogen associated with the most severe symptoms (McIntire 2023). Additionally, highly symptomatic leaves have been observed to senesce earlier in the fall (Fearer et al. 2022; McIntire and Vieira 2025). Given the observed shifts in leaf traits associated with BLD, in tandem with the dominant nature of beech trees in many forests, the disease may alter litter decomposition rates within infested forests. That said, the impacts of BLD on rates of leaf litter decomposition have yet to be studied.

Litter decomposition is a key ecosystem process, releasing nutrients back into forested ecosystems (Cain et al. 2014). In deciduous forests, a large quantity of detritus consists of leaves fallen from the surrounding tree canopy, accounting for upwards of 70% of aboveground litterfall (Karberg et al. 2008). The leaf litter in forests acts as a protective layer for upper soil layers, reducing soil drying, minimizing temperature fluctuations, and limiting erosion associated with precipitation events (Ginter et al. 1979; Lowdermilk 1930; Sayer 2006). The litter layer on the forest floor is also critical habitat for many organisms including invertebrates, bacteria, and fungi (Johnson and Catley 2002). Decomposition is a key regulator of carbon (C) cycling (Karberg et al. 2008). Fluctuations in litter decomposition can reduce carbon storage on the forest floor, releasing carbon stored in organic matter back into the atmosphere (Hicke et al. 2012; Norman and Kreye 2023). Shifts in typical decomposition processes could ultimately influence the global carbon cycle, which has already seen major changes driven by anthropogenic influences (May Ver et al. 1999; Pongratz et al. 2009). In addition to carbon cycling, decomposition is vital in nutrient cycling. The breakdown of leaf litter makes nutrients like nitrogen (N), phosphorus (P), and calcium (Ca) available for uptake by trees and other vegetation (Cain et al. 2014). Changes in rates of litter decomposition can alter the speed at which nutrients are made available.

Litter traits can be strong drivers of decomposition. Forest diseases that alter leaf characteristics can ultimately influence leaf litter and may be particularly impactful on decomposition rates within ecosystems (Cobb and Rizzo 2016). Both chemical and physical traits of leaves play important roles in determining decomposition. Higher ratios of C:N in leaf litter are linked to slower release of nutrients during decomposition (Bani et al. 2018; Cain et al. 2014). Decomposition is similarly slowed by high lignin:N ratios (Taylor et al. 1989). Furthermore, SLA is typically positively correlated with decomposition (Rosenfield et al. 2020; Zukswert and Prescott 2017). Multiple studies have documented changes in leaf traits as a result of BLD (such as shifts in SLA and leaf N concentration). Therefore, this disease is likely to alter litter quality in ways that may have important impacts on litter decomposition.

Decomposition is further influenced by the biotic environment, with fungi considered to be critical decomposers of leaf litter (Schneider et al. 2012). As such, the specific taxonomic composition and diversity of fungal decomposers can affect decomposition rates (reviewed in van der Wal et al. 2013). The composition of the fungal community can be driven by a wide range of environmental factors (Aneja et al. 2006; Glassman et al. 2018; Prescott and Grayston 2013), including the specific makeup of the decomposing organic matter (Hobbie et al. 2012; Swan and Palmer 2006). As such, it seems likely that the fungal communities that are key to litter decomposition could be influenced by forest diseases, particularly foliar diseases. Though fungal communities have not yet been explored during litter decomposition in the case of BLD, the disease has been shown to drive changes in fungal communities on living leaves (Burke et al. 2024). During decomposition, fungal communities tend to progress from taxa that readily decompose more labile compounds, such as cellulose, to those that are capable of decomposing recalcitrant fractions, such as lignin (van der Wal et al. 2013). That said, initial colonization by fungal taxa well suited to the particular makeup of the litter tends to lead to the fastest rates of litter decay (Cline and Zak 2015).

Here, we explore the impacts of beech leaf disease on litter decomposition using a field‐based litterbag study to quantify rates of decomposition in BLD‐symptomatic and asymptomatic leaves. We hypothesized that BLD would alter rates of litter decomposition due to changes in leaf anatomy and chemistry, such as increased thickness and decreased SLA, and increased foliar nitrogen. To further understand possible drivers of changes in litter decomposition, we also investigated fungal communities associated with decomposing beech litter. Since BLD is documented to alter fungal communities on living leaves, we hypothesized that fungal communities would also differ between symptomatic and asymptomatic leaf litter.

## 2 Methods

### 2.1 Litter Acquisition

In October 2023, freshly senesced American beech leaves were collected from 16 trees selected haphazardly from a beech orchard at the Holden Arboretum in Kirtland, OH, USA (41.611447, −81.300192). The orchard was originally established in 2006 to explore resistance to another significant disease: Beech Bark Disease. Trees within this orchard are not affected by BBD. Within the plantation, trees currently exhibit a range of BLD severity, making it possible to collect both symptomatic and asymptomatic litter from trees of similar ages growing under the same conditions. We separated leaves into two composite sample groups based on visual symptom expression. Asymptomatic leaves were classified as those with no visually symptomatic leaf tissue. Symptomatic leaves were classified as those with symptoms (darkened and curling) impacting more than 75% of the leaf. Leaves were dried at 60°C for 48 h until weight remained constant. Approximately 3.5 g of whole leaf litter of either symptomatic or asymptomatic leaves was placed in each of the 160 litterbags (21 cm × 14.5 cm). Bags were constructed using nylon window screen with a mesh size of 1.5 × 0.5 mm, allowing for soil micro‐ and meso‐fauna to enter the bags, as well as fungal and bacterial colonizers.

Excess leaves collected for the litterbags were used for nutrient analysis. We ground approximately 3 g of asymptomatic leaves and 3 g of symptomatic leaves using a coffee and spice grinder (Stanley Black & Decker, New Britain, CT, USA), followed by a mortar and pestle until the tissue was less than 1 mm in size. Three samples of each symptom expression (1 g each) were sent to Pennsylvania State University Agricultural Analytical Services Laboratory (University Park, PA, USA) for total nitrogen (N) and carbon (C) dry combustion analysis. Both the total N concentration and total C concentration were averaged across the three samples for both the symptomatic and asymptomatic leaves separately. A two‐sample *t*‐test was used to compare the means of C concentration, N concentration, and C:N ratio.

### 2.2 Field Decomposition Trial

On November 1, 2023, we deployed the litterbags in two forests located at the Holden Arboretum: Baldwin Forest and Bole Woods. Both forests are naturally occurring beech‐maple forests dominated by American beech ( *Fagus grandifolia* ), sugar maple ( *Acer saccharum* ), and tulip poplar ( *Liriodendron tulipifera* ). Both sites, located within 3.5 km of the litter acquisition site, were unmanaged and included mature (≥ 12.7 cm DBH) American beech trees in the canopy. As both sites are located near the epicenter of BLD, American beech at each site is, in general, highly symptomatic and has been expressing symptoms of BLD for over a decade. Beech bark disease is not present at either site. The region is characterized by warm humid summers and cold snowy winters. The average temperature for the study period (November 2023–October 2024) was 11.3°C and total rainfall was 103.4 cm (“NOWData” 2024).

At each site, we cleared away the naturally occurring leaf litter, exposing the organic soil layer. We placed bags flush with the ground and anchored them in place using ground staples. The previously removed litter layer was then used to fully cover the litterbags. Bags were collected at four 12‐week intervals beginning on January 24, 2024, and ending on October 2, 2024. Two plots were established at each site roughly 10 m apart (2 sites × 2 plots × 2 symptom expression categories × 4 time intervals × 5 replicates = 160 leaf litterbags). Bags were placed in parallel columns approximately 12.7 cm apart, grouped by symptom expression and time interval. One leaf litterbag was never recovered from the field (symptomatic litter in Baldwin Forest at the 48‐week interval); therefore, the final leaf litterbag count was 159. When removed from the field, we gently cleaned the bags to remove excess dirt. Bags were then dried at 60°C for 48 h and weighed to determine dry mass.

#### 2.2.1 Statistical Analysis

We calculated percent decomposition for each litterbag using the following equation: ([initial dry weight − final dry weight]/initial dry weight) * 100. Initially, a two‐way analysis of covariance (ANCOVA) was conducted testing percent decomposition as a function of symptom expression, time, site, and the interaction between symptom expression and site. Since the interaction between symptom expression and site was not significant, the interaction term was removed from the final analysis.

We estimated rates of decomposition for symptomatic and asymptomatic litter at each site separately using the following exponential decay model: *M*_*t*_ = *M*_0_ * *e* ^−*kt*^, where *M*_*t*_ is mass at time *t*, *M*_0_ is initial mass and *k* is the decay constant indicating the rate of decomposition (Bärlocher 2005). We rewrote the equation in a linear format as: ln[*M*_*t*_] = ln[*M*_0_*e ^−*kt*^] = ln[*M*_0_] − *kt*, then regressed the natural log of *M*_*t*_ as a function of time. The regression was then used to estimate the slope, or *k*, for each symptom expression and site. 95% confidence intervals were calculated to determine significance (see Bärlocher 2005). We determined confidence intervals by multiplying the standard error of the slope estimate by 1.96.

### 2.3 Microbial Community

#### 2.3.1 DNA Extraction, PCR, and High‐Throughput Sequencing

After removal from the field, we collected approximately 0.1 g of the leaf litter using sterile forceps from each litterbag for microbial analysis. We gently tore the samples apart using sterile forceps before lysing the samples in bead‐beating tubes containing 300 mg of 400 μM sterile glass beads (VWR, West Chester, PA, USA) and 200 mg of 1 mm sterile glass beads (Chemglass, Vineland, NJ, USA). Bead beating was done with a Precellys homogenizer (Bertin, Technologies, France) for 40 s in 2% CTAB (cetyltrimethyl ammonium bromide). We extracted DNA with a phenol–chloroform method, precipitated with 20% polyethylene glycol 8000, and desalted using 80% EtOH (Burke et al. 2019). We dried and suspended DNA in 100 μL TE (Tris EDTA) buffer following precipitation. DNA was stored in a 1.5 mL low‐retention microcentrifuge tube (Fisher Scientific, Waltham, MA, USA) at −20°C until analysis. To amplify fungal DNA, we used fungal primers 58A2F (ATCGATGAAGAACGCAG; Martin and Rygiewicz 2005) and ITS4 (TCCTCCGCTTATTGATATGC; White et al. 1990) to amplify the ITS 2 region. The primers contained Illumina overhang adapters for downstream sequencing (as in Burke et al. 2024). PCR reactions included 1 unit FastStart Taq DNA polymerase (Sigma Aldrich, Saint Louis, MO, USA), 2 mM MgCl_2_, 0.8 mM of dNTPs, 0.2 μM of each primer, 0.5 μg/μL bovine serum albumin, and 1 μL of leaf DNA template in 25 μL total volume. We included negative controls in all PCR runs to account for possible fungal DNA contamination. PCR was completed on a S1000 thermal cycler (Bio‐Rad Inc. Hercules, CA) with the following conditions: an initial denaturation for 5 min at 95°C, followed by 30 cycles of denaturation for 30 s at 95°C, annealing for 1 min at 60°C, and extension for 1 min at 72°C, with a final extension of 5 min at 72°C. We confirmed positive amplification with gel electrophoresis. Samples were sequenced at Case Western Reserve University Genomics Core Laboratory (Cleveland, OH, USA) on an Illumina MiSeq V3 sequencer with a 2 × 250 bp run. PCR products with low yield (4 total) were not included in the sequencing run (155 total samples were run with Illumina MiSeq).

#### 2.3.2 Bioinformatics Pipeline

A total of 19.4 million fungal reads were yielded in our sequencing effort, with one sample being removed due to low yield (fewer than 500 reads). We processed a total of 154 leaf samples with the UNOISE pipeline (Edgar 2016). We used the program USEARCH (version 11.0.667; Edgar 2010) to merge forward and reverse sequence reads with the *fastq_mergepairs* command and remove control phiX reads with the *filter_phiX* command. Reads were then trimmed of PCR primers using Cut Adapt (v2.8; Martin 2011) where we allowed up to three mismatches across the length of each primer. We then used USEARCH to remove short reads (less than 250 bp) that had one or more sequencing errors (f*astq_filter* command) and ensured all reads were in the same orientation (*orient* command against the UNITE database). The filtered and oriented reads were then used to create error‐corrected and chimera‐filtered sequence variants (i.e., zero radius OTUs or zOTUs) with the *unoise3* command, where zOTUs with fewer than eight sequence reads were removed (per the default settings). We then mapped the merged reads from each leaf sample with control phiX and primers removed to the zOTUs with the *otutab* command. We made taxonomic assignments for the zOTUs with the SINTAX algorithm (Edgar 2016) by comparing against the UNITE fungal database (version 10.0 release date 2024‐04‐04; Abarenkov et al. 2024). This resulted in 4320 fungal zOTUs, which were further assigned to functional guilds using the FUNGuild parser (v1.2; Nguyen et al. 2016). Only guilds considered “probable” or “highly probable” assignments were retained. We determined the average relative abundance of four fungal guilds (mycorrhiza, epiphytes, plant pathogens, and saprotrophs) for each sample by summing the abundance of the retained zOTUs for each guild and dividing by total zOTU abundance per sample. While some fungal taxa are known to display multiple guilds and can change function depending on life stage or environmental conditions (Nguyen et al. 2016), assigning taxa to guilds allowed us to explore the potential functions of the taxa present in the litterbags.

#### 2.3.3 Statistical Analysis of Microbial Communities

We analyzed fungal community composition for each removal interval (12, 24, 36, and 48 weeks). To account for different sequencing depths between samples, we first normalized the raw sequence read counts (McMurdie and Holmes 2014) with the *estimateSizeFactors* function in the package DESeq2 (version 1.36.0; Love et al. 2014). We analyzed differences in fungal community composition between symptomatic and asymptomatic litter and between the two sites using permutational multivariate analysis of variance (PERMANOVA) and permutational multivariate analysis of dispersion (PERMDISP) in the vegan R package (version 2.6‐8; Oksanen et al. 2013). These analyses used Bray–Curtis dissimilarity matrices, which we calculated with the *vegdist* function and “method = bray” option. We ran four PERMANOVAs (one for each removal interval; 12, 24, 36, and 48 weeks) using the *adonis2* function and allowing 4999 permutations, with site, symptom expression, and their interaction as explanatory variables. The function *betadisper* was used for PERMDISP, followed by a permutation test using the *permutest* function and 999 permutations to test for multivariate dispersion. We used NMDS to visualize differences in community composition among groups with the function *metamds*. We compared the relative abundances of each of the four fungal guilds as a function of symptom expression using Wilcoxon Rank sum tests with continuity correction.

## 3 Results

### 3.1 Leaf Traits

Total C was similar between asymptomatic and symptomatic leaves (*t* = −1.14, df = 4, *p* = 0.32) while total N was significantly higher in symptomatic leaves relative to asymptomatic leaves (*t* = −6.49, df = 4, *p <* 0.01; Table 1). As a result, symptomatic leaves had a lower C:N ratio than asymptomatic leaves (*t* = 9.15, df = 4, *p* < 0.01; Table 1).

**TABLE 1:** Nutrient concentrations and carbon: nitrogen ratio (mean ± SE, *n* = 3) for each symptom expression.

Symptom Expression	Carbon (%)	Nitrogen (%)*	Carbon: Nitrogen*
Asymptomatic	48.90 ± 0.06	0.68 ± 0.01	71.90 ± 0.31
Symptomatic	49.20 ± 0.15	0.83 ± 0.01	59.60 ± 0.71

*Note:* Factors that differ significantly between asymptomatic and symptomatic litter (*p* < 0.01) are denoted with an asterisk.

### 3.2 Decomposition

**FIGURE 1:**
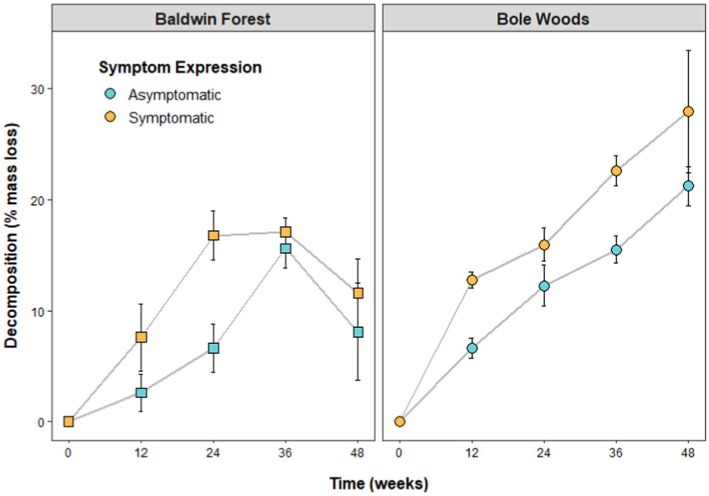
Mean percent decomposition (±SE) through time, by site and symptom expression.

**FIGURE 2:**
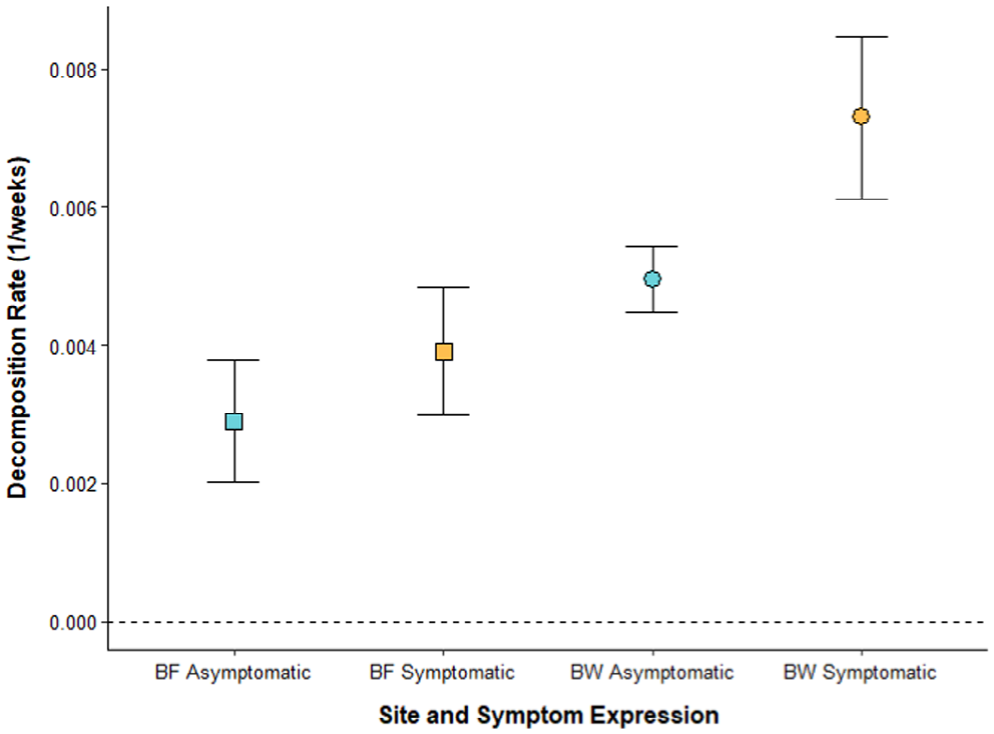
Decomposition rates (±95% confidence intervals, *n* = 40) by symptom expression for Baldwin Forest (BF) and Bole Woods (BW).

Symptomatic litter decomposed more than asymptomatic litter (*F*_1,155_ = 18.10, *p* < 0.01), when accounting for variability across time (*F*_3,155_ = 35.62, *p* < 0.01; Figure 1) and site (*F*_1,155_ = 21.87, *p* < 0.01; Figure 1), both of which were also significant drivers of decomposition. As such, rates (*k*, 1/weeks) differed between symptomatic and asymptomatic litter and between sites. Symptomatic litter decomposed at a significantly higher rate than asymptomatic litter in Bole Woods (based on nonoverlapping 95% confidence intervals; Figure 2). That said, while decomposition rates were also consistently higher on symptomatic litter relative to asymptomatic litter in Baldwin Forest, these differences were not significant late in decomposition (Weeks 36 and 48; Figures 1 and 2). Decomposition rates were faster at Bole Woods relative to Baldwin Forest across both symptom expressions (Figure 2). Notably, the litterbags at Baldwin Forest that were removed after 48 weeks showed a lower proportional mass loss than those at 36 weeks (Figure 1). A possible explanation may be the accumulation of soil and/or biomass from fungal or surrounding plant growth, which may have been overlooked while litterbags were being processed (Karberg et al. 2008; Krishna and Mohan 2017).

### 3.3 Microbial Community Analysis

**FIGURE 3:**
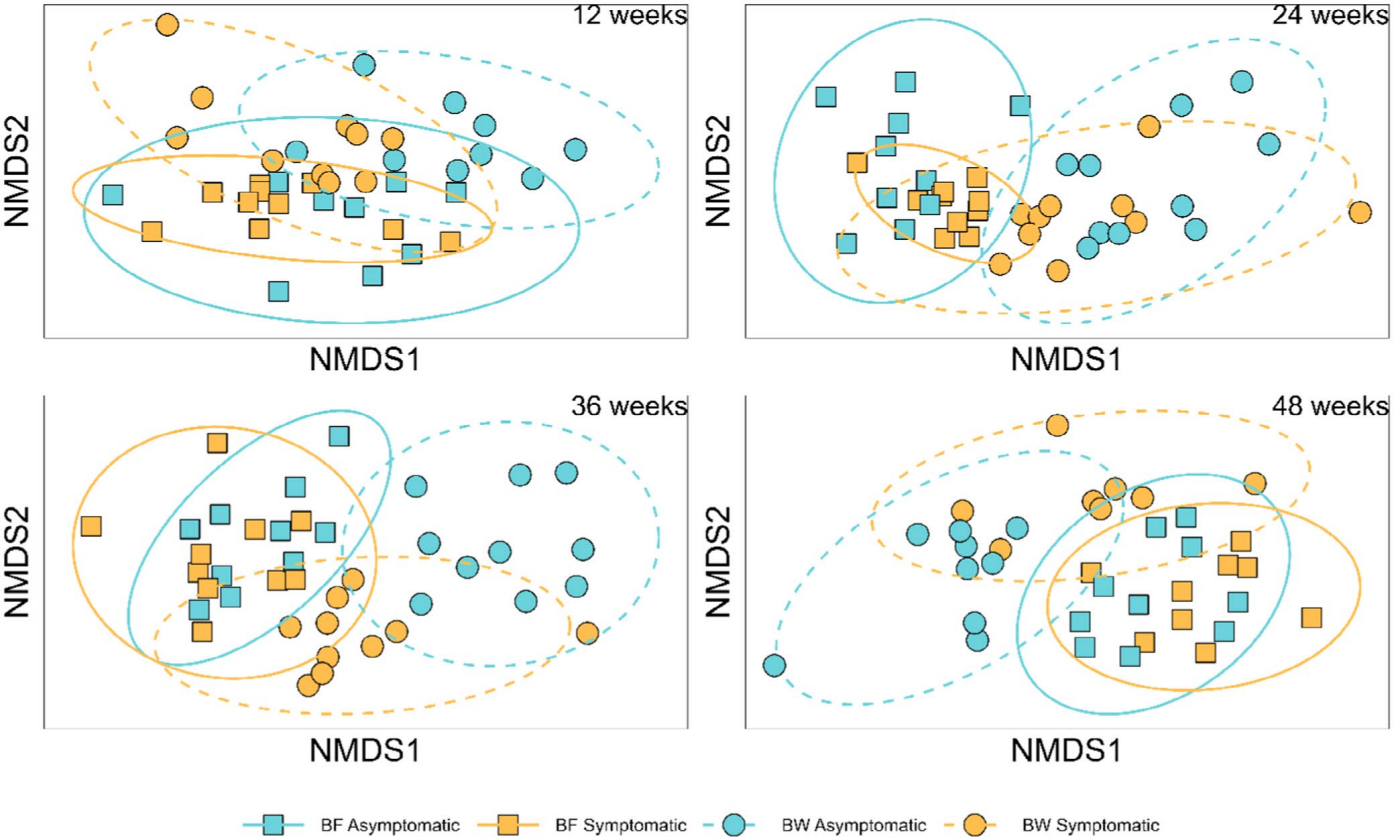
Two‐dimensional nonmetric multidimensional plots based on Bray–Curtis dissimilarity by site and symptom expression for each collection time. Ellipses represent 95% confidence intervals based on standard deviation. Ordination stress across two dimensions was as follows: 12 weeks = 0.22; 24 weeks = 0.19; 36 weeks = 0.17; 48 weeks = 0.18.

Fungal community composition differed across symptom expression and site at all four time points (Table 2, Figure 3). Symptom expression and site interacted significantly at the 36‐ and 48‐week time points (Table 2, *p* < 0.05).

**TABLE 2:** PERMANOVA and PERMDISP results, indicating differences in community composition by symptom expression, site, and their interaction, separately for each time point.

adonis2 (formula = dis ~ symptom expression * site, data, permutations = 4999, by = “terms”)					
	df	Sum of Sqs	*R* ^2^	*F*	*p*
12 weeks					
Symptom Expression	1	0.559	0.083	3.496	< 0.001***
Site	1	0.606	0.089	3.784	< 0.001***
Symptom Expression: Site	1	0.165	0.024	1.032	0.393
Residual	34	5.441	0.804		
Total	37	6.771	1.000		
Dispersion—Site	1	0.003		0.5802	0.451
Dispersion—Symptom Expression	1	0.013		2.079	0.152
24 weeks					
Symptom Expression	1	0.788	0.079	3.686	< 0.001***
Site	1	1.282	0.129	5.996	< 0.001***
Symptom Expression: Site	1	0.209	0.021	0.976	0.441
Residual	36	7.699	0.772		
Total	39	9.979	1.000		
Dispersion—Site	1	0.015		2.242	0.15
Dispersion—Symptom Expression	1	0.071		12.745	0.002**
36 weeks					
Symptom Expression	1	0.900	0.065	3.061	< 0.001***
Site	1	1.703	0.124	5.790	< 0.001***
Symptom Expression: Site	1	0.569	0.041	1.935	< 0.001***
Residual	36	10.588	0.769		
Total	39	13.761	1.000		
Dispersion—Site	1	0.010		3.808	0.052
Dispersion—Symptom Expression	1	0.008		2.881	0.094
48 weeks					
Symptom Expression	1	0.538	0.037	1.459	0.039*
Site	1	1.531	0.106	4.152	< 0.001***
Symptom Expression: Site	1	0.578	0.040	1.567	0.026*
Residual	32	11.799	0.817		
Total	35	14.446	1.000		
Dispersion—Site	1	0.001		0.493	0.497
Dispersion—Symptom Expression	1	0.000		0.252	0.616

*Note:* Statistical differences are indicated by the following: *p ≤ 0.05, **p ≤ 0.01, ***p ≤ 0.001.

**FIGURE 4:**
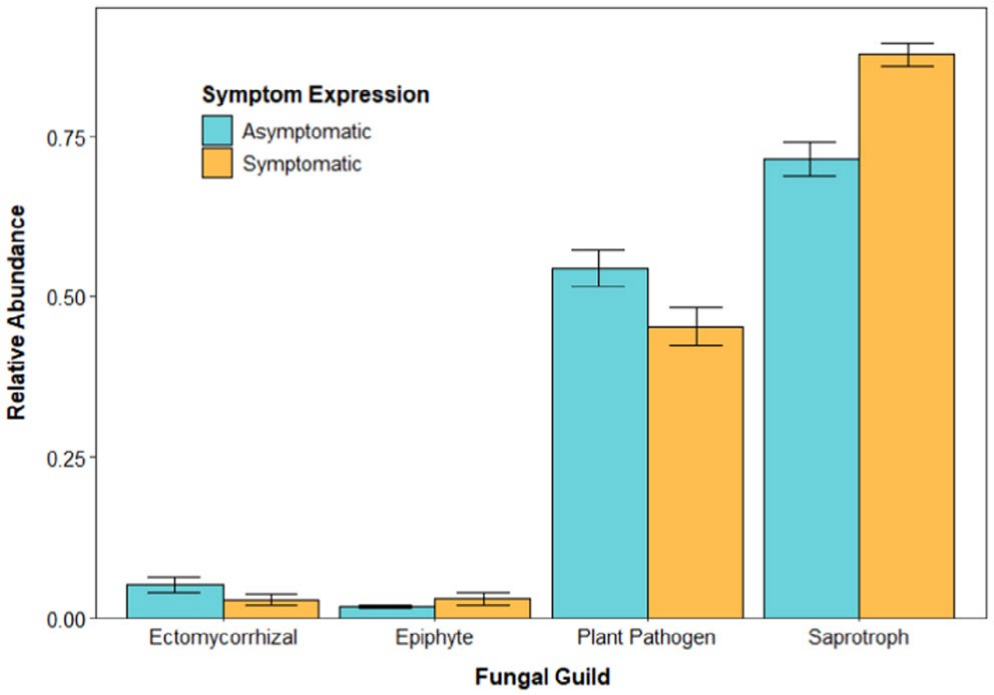
Relative abundance (mean ± SE, *n* = 154) of the four fungal guilds by symptom expression, pooled across sites and collection times.

Saprotrophs (decomposers) constituted the most abundant fungal guild on both symptomatic and asymptomatic litter. Furthermore, saprotrophs were more abundant on symptomatic litter than asymptomatic litter (*W* = 1581, *p* < 0.01; Figure 4). Relative abundances of the ectomycorrhizal and epiphyte fungal guilds were similar (and relatively low) on the decomposing litter of symptomatic and asymptomatic leaves (ectomycorrhizal: *W* = 3399, *p* = 0.12; epiphyte: *W* = 2908, *p* = 0.84). Asymptomatic litter had a higher relative abundance of fungal plant pathogens than symptomatic litter (*W* = 3572, *p* = 0.03; Figure 4). The relative abundance of fungal guilds varied across the two sites (Figure S1). For specific information on the specific fungal genera present, see Figures S1 and S3.

## 4 Discussion

Our findings suggest BLD may influence important ecosystem processes such as litter decomposition. Specifically, we found that BLD‐symptomatic leaf litter decomposed more quickly than asymptomatic litter. These changes were associated with shifts in leaf traits and the associated fungal community, both of which have the capacity to be strong drivers of decomposition. Critically, faster decomposition of BLD‐symptomatic leaves could result in increased rates of nutrient and carbon cycling.

Differences in decomposition rates can be partially attributed to BLD‐driven changes in leaf traits. In particular, increased foliar N concentration is a strong driver of increased rates of litter decomposition (Melillo et al. 1982; Zhang et al. 2008). Our findings of increased foliar N in symptomatic leaves align with the findings of others suggesting that BLD can increase N levels within beech leaves (McIntire 2023). Increases in N levels have been similarly associated with other forest pathogens. For example, *Ulmus minor* (field elm), which is highly susceptible to *Ophiostoma novo‐ulmi* (a pathogen responsible for Dutch elm disease), was found to have higher N concentrations in highly symptomatic leaves, resulting in a low C:N ratio and ultimately increased decomposition (Ferreira et al. 2022). That said, the relationship between plant disease and foliar nutrients is complex and often inconsistent across tree species and forest pests (Ferreira et al. 2022; Huber et al. 2011). Recent research has also shown BLD to result in thicker leaves with lower specific leaf areas (Colbert‐Pitts et al. 2025; Fletcher et al. 2024). This change also has the potential to alter rates of decomposition. In general, leaves with low SLA tend to decompose more slowly relative to leaves with higher SLA when exploring across species (e.g., Bakker et al. 2011; Zukswert and Prescott 2017). Symptomatic beech leaves showed increased rates of decomposition, suggesting that shifts in plant traits (like the increase in foliar N) are likely important drivers of decomposition in this case. It should be noted that litter was oven dried prior to nutrient analysis and being deployed in the field. Oven drying at temperatures above 60°C has been documented to alter the chemical composition of leaves such as reducing total N concentration (Ramsumair et al. 2014). Notably, however, this treatment was applied consistently to both symptom expressions.

In addition to leaf traits themselves, another critical driver of decomposition is the microbial community. Fungi, in particular, serve as important catalysts of decomposition (Krishna and Mohan 2017; Schneider et al. 2012). We found significant differences in microbial communities between symptomatic and asymptomatic litter, and these differences persisted through time. Importantly, saprotrophic fungi were more abundant on symptomatic litter as compared to asymptomatic. Saprotrophs are the main functional guild of decomposers; therefore, a higher abundance in symptomatic leaf tissues logically coincides with increased decomposition (Niego et al. 2023). Differences in fungal communities between symptom expressions could be partially explained by the increased foliar N in symptomatic leaves. An important element for microbial growth and reproduction, N concentrations can be a strong driver of fungal communities (Song et al. 2023) and litter with low C:N ratios is often preferentially colonized (Song et al. 2023). Differences in fungal communities on decomposing beech litter could also be driven, in part, by differences in fungal communities on symptomatic and asymptomatic live leaves. Indeed, the fungal community associated with live beech leaves (the phyllosphere) has been shown to be altered both by the presence of *L.crenata mccannii* and BLD symptoms (Burke et al. 2024). If these community differences persist in senesced leaves, this could have cascading effects on decomposition and carbon storage in forests. There is evidence from other systems that the trajectory of fungal community shifts during decomposition can be driven by the initial fungi colonizing the decaying material (Cline and Zak 2015; Fukami et al. 2010; Hiscox et al. 2015; van der Wal et al. 2013), with fungi from the phyllosphere becoming important members of the decomposer community, at least for the first few months (Osnono 2006; Voříšková and Baldrian 2013). It should be noted that the leaves used in the current study were oven dried prior to field deployment. While not dried at a temperature or length of time to kill all fungi (see Gleason et al. 2004; Niyogi et al. 2020), oven drying in the current study likely altered how phyllosphere taxa interacted with soil communities during decomposition (Fanin et al. 2021). Linking the cascading effects of BLD‐induced changes to phyllosphere communities on forest ecosystem function is an interesting avenue for future research.

BLD‐driven changes in litter decomposition rates may alter ecosystem structure and function by altering important processes such as carbon cycling. Faster decomposition of beech litter associated with BLD may result in temporary surges of released C, and a slightly reduced capacity of these forests to store C. Even more consequential may be the combination of increased decomposition rates with the likely reduction of beech litter. Logically, BLD is likely to reduce beech litter inputs through a combination of increased tree mortality (Shepherd et al. 2025) and canopy thinning from early leaf drop and bud abortion (Fearer et al. 2022; Martin and Volk 2022). Increased mortality and premature senescence associated with BLD may cause fluctuations in leaf litter quantity in heavily infested areas. Not only could these shifts lead to a reduced capacity of infected forests to sequester carbon, but it could also lead to shifts in nutrient cycling within temperate forests. Increased availability of nutrients, including N, P, Ca, and magnesium (Mg) can influence forest community composition (Edmonds et al. 1989). N is considered a limiting nutrient in many ecosystems. Important for plant survival, large changes in N availability can promote plant growth, but in some cases, have led to declines in biodiversity (Bernhard 2010). Additionally, P has been shown to be a growth‐limiting nutrient in temperate forests due to a legacy of acid deposition (Jahn et al. 2025). Nutrient availability in forests is largely influenced by canopy characteristics (Prescott 2002). More readily decomposing leaf litter due to BLD could potentially create temporary increases in nutrient availability followed by a reduction in nutrient inputs as canopies thin and trees die in highly infested areas. Major fluctuations in nutrient availability could exacerbate changes to forest community composition from BLD‐related mortality. The dominance of beech (Tubbs and Houston 1990) drives the potential of BLD to have an outsized impact on carbon dynamics and nutrient cycling across large geographic scales.

Other forest pathogens have also been linked to altered decomposition rates, though the direction and magnitude of such shifts can be tree and pest specific. Similar to our findings associated with BLD, *Alnus lusitanica* (Iberian alder) litter symptomatic for *Phytophthora* x*alni* and *Ulmus minor* (field elm) symptomatic for *Ophiostoma novo‐ulmi* were found to decompose more quickly (Ferreira et al. 2022). Inversely, leaf litter of *Castanea sativa* (sweet chestnut) impacted by *Phytophthora cinnamomic* has been shown to decompose more slowly. That said, disease does not always shift rates of litter decomposition. *Phytophthora ramorum* (the cause of sudden oak death) did not alter rates of litter decomposition for either *Notholithocarpus densiflorus* (tanoak) or *Umbellularia californica* (California bay laurel; Cobb and Rizzo 2016). However, at the ecosystem level, the disease did shift rates of carbon and nutrient cycling by driving shifts in the overstory tree composition (Cobb and Rizzo 2016). Rates of litter decomposition for *Pinus densiflora* (Japanese red pine) were consistent from forests lightly impacted by pine wilt disease to highly impacted forests (Kim et al. 2019). Similarly, we might expect the ecosystem‐level consequences of BLD to continue to intensify as mortality of American beech and subsequent changes in forest composition become more widespread. Such disease‐driven shifts can be quite impactful. In some cases, forest pathogen‐driven reductions in net primary productivity combine with increases in decomposition to reduce net ecosystem productivity (Hicke et al. 2012). In the case of severe outbreaks, such shifts have the potential to switch stands from C sinks to sources (Hicke et al. 2012).

Litter decomposition is further modified by the environment. Abiotic conditions, such as temperature, moisture, and pH can influence decomposition, both directly, as well as through their influence on microbial communities. These conditions are strongly influenced by larger‐scale geographic patterns, as well as microsite conditions. Unsurprisingly, we observed different patterns of both decomposition and microbial communities across the two forested sites. These results suggest that while we expect BLD to speed litter decomposition, the magnitude of such changes will almost certainly vary from forest to forest (or even within forests). Further, we might expect these dynamics to shift over time as BLD progresses, likely opening the canopy and increasing both temperature and throughfall in the forest understory, both of which can influence rates of decomposition (Cain et al. 2014).

## 5 Conclusion

Increased rates of litter decomposition as a result of beech leaf disease incidence have the potential to alter nutrient and carbon cycling in forest systems. These changes are likely underpinned by a combination of changes in both foliar characteristics (such as C:N ratio) and in associated fungal communities. As BLD spreads across the northeastern United States, and beyond, we might expect these shifts in litter decomposition to interact with changes in litter inputs, via a combination of decreased leaf production and tree mortality, to drive even bigger shifts in forest nutrient and carbon budgets. While there are still many unknowns regarding the full extent to which BLD will alter forest ecosystems, this study provides crucial first insights into some of the pathways by which BLD is poised to alter the functioning of many forests.

## Author Contributions

**Brianna L. Shepherd:** conceptualization (lead), data curation (lead), formal analysis (equal), investigation (lead), methodology (lead), writing – original draft (lead). **Sarah R. Carrino‐Kyker:** data curation (supporting), formal analysis (equal), investigation (supporting), writing – review and editing (supporting). **David M. Jenkins:** data curation (supporting), formal analysis (equal), investigation (supporting), writing – review and editing (supporting). **David J. Burke:** conceptualization (supporting), funding acquisition (equal), investigation (supporting), methodology (supporting), writing – review and editing (supporting). **Katharine L. Stuble:** conceptualization (supporting), funding acquisition (equal), investigation (equal), project administration (lead), writing – review and editing (equal).

## Funding

This research is funded by the Nature Conservancy's Forest Pest and Pathogen Program, through their Tree Species in Peril program, and by the Holden Foundation.

## Conflicts of Interest

The authors declare no conflicts of interest.

## Supporting information


**Data S1:** ece372563‐sup‐0001‐supinfo.docx.

## Data Availability

Sequence data are available through the Sequence Read Archive of Genbank under BioProject number PRJNA1291822. All other required data are uploaded as Data S1.
