# Supplementary material for: Beech Leaf Disease Associated With Changes in Litter Decomposition and Fungal Communities
Source: Ecol Evol. 2025 Nov 29;15(12):e72563. doi: 10.1002/ece3.72563 (PMC12664823; doi:10.1002/ece3.72563)
Supplement: Supplementary file 1 — Data S1: ece372563‐sup‐0001‐supinfo.docx. [file ECE3-15-e72563-s001.docx]

**6. Appendix**

**
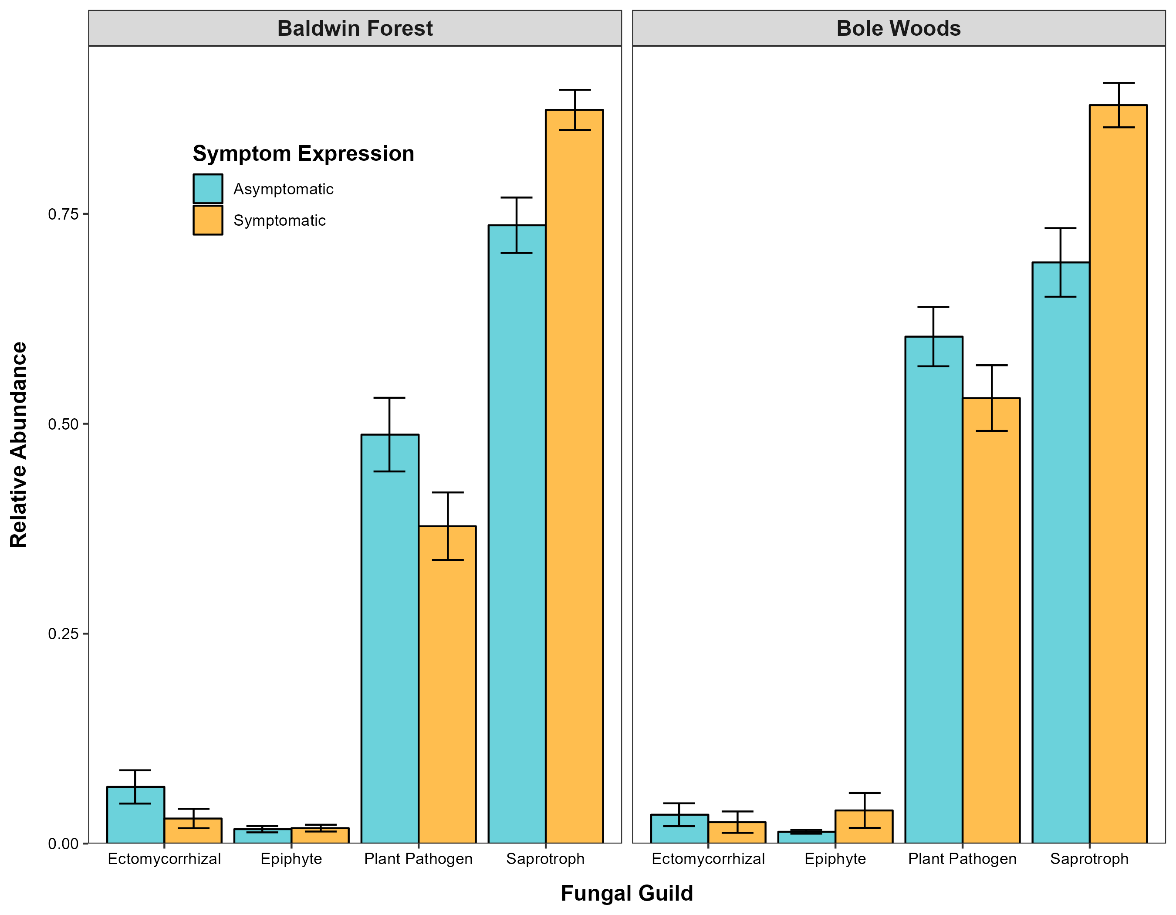
**

**Figure A.1.** Relative abundance (mean ± standard error, n =1475) of the four fungal guilds by symptom expression and site.

To compare average relative abundance of each of the four fungal guilds as a function of site and symptom, we used a Kruskal-Wallis rank sum test followed by a Wilcoxon rank sum exact test. The average relative abundance for both ectomycorrhizal and epiphyte fungal guilds did not differ by site or symptom expression (ectomycorrhizal: χ^2^ = 2.81, df = 3, *p* = 0.42; epiphyte: χ^2^ = 0.44, df = 3, *p* = 0.93). Significant differences in plant pathogen abundance were primarily between the two forests for symptomatic litter. Symptomatic litter at Baldwin Forest had significantly lower pathogen abundance than both asymptomatic (*p* < 0.01) and symptomatic litter (*p* = 0.011) at BW. The average relative abundance of saprotrophs was higher in symptomatic litter across all sites and symptoms (Figure A.1). Asymptomatic litter had statistically lower abundances of saprotrophs than symptomatic litter (χ^2^ = 25.39, df= 3, *p* < 0.01) at BF. Similarly, asymptomatic litter also had lower abundances of saprotrophs than symptomatic litter (*p* < 0.01) at Bole Woods.

**
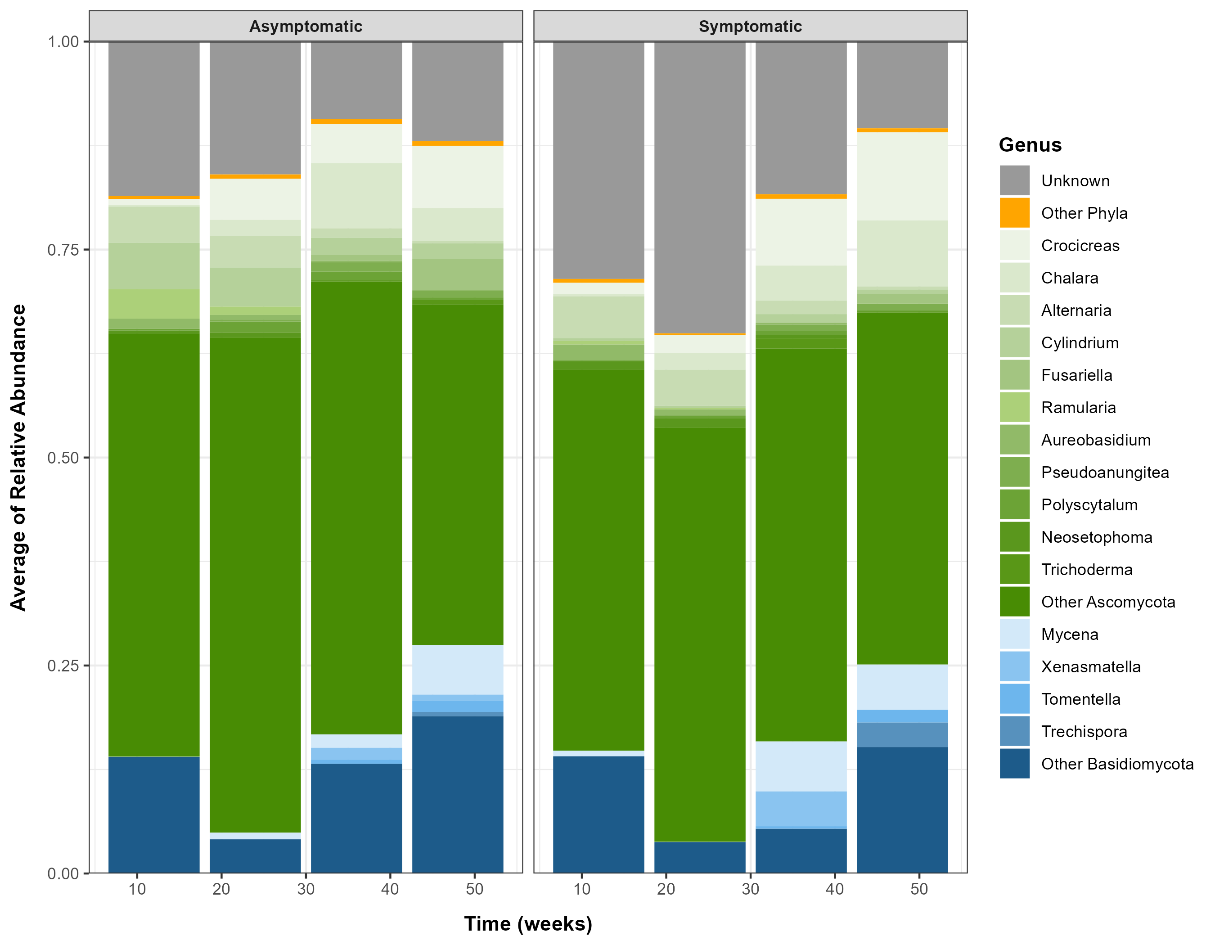
**

**Figure A.2**. Average relative abundance of the fifteen most abundant fungal genera by symptom expression for each sampling period. Note – Identification of zOTUs was completed using the UNITE database. In our samples, the genus *Chalara* was identified, but not to species. It should be noted that a number of *Chalara* species are anamorphs of other genera in the Ascomycota phylum. For example, *Chalara fraxinea,* has been identified as the asexual stage of *Hymenoscyphus fraxineus*.


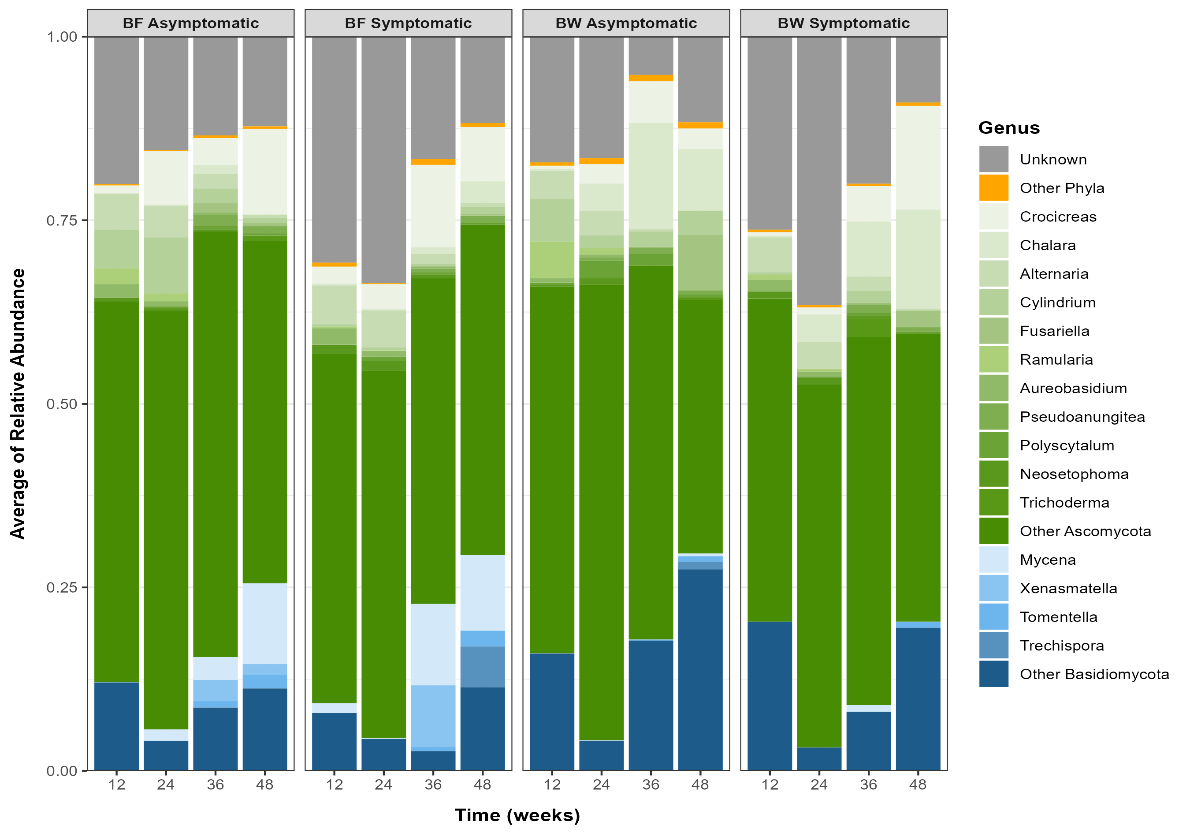


**Figure A.3**. Average relative abundance of the fifteen most abundant fungal genera by symptom expression for each sampling period in each forest.
